# The Labdane Ent-3-Acetoxy-Labda-8(17), 13-Dien-15-Oic Decreases Blood Pressure In Hypertensive Rats

**DOI:** 10.5935/abc.20160058

**Published:** 2016-06

**Authors:** Janaina A. Simplicio, Marilia R. Simão, Sergio R. Ambrosio, Carlos R. Tirapelli

**Affiliations:** 1 Programa de Pós-Graduação em Farmacologia - Faculdade de Medicina de Ribeirão Preto, Universidade de São Paulo (USP), Ribeirão Preto, SP - Brazil; 2 Núcleo de Pesquisa em Ciências e Tecnologia - Universidade de Franca (UNIFRAN), Franca, SP - Brazil; 3 Departamento de Enfermagem Psiquiátrica e Ciências Humanas - Laboratório de Farmacologia - Escola de Enfermagem de Ribeirão Preto (USP), Ribeirão Preto, SP - Brazil

**Keywords:** Labdane, Vascular Relaxation, Diterpene, Forskolin, Renovascular Hypertension

## Abstract

**Background:**

Labdane-type diterpenes induce lower blood pressure via relaxation of vascular smooth muscle; however, there are no studies describing the effects of labdanes in hypertensive rats.

**Objective:**

The present study was designed to investigate the cardiovascular actions of the labdane-type diterpene ent-3-acetoxy-labda-8(17), 13-dien-15-oic acid (labda-15-oic acid) in two-kidney 1 clip (2K-1C) renal hypertension.

**Methods:**

Vascular reactivity experiments were performed in aortic rings isolated from 2K-1C and normotensive (2K) male Wistar rats. Nitrate/nitrite (NOx) measurement was performed in aortas by colorimetric assay. Blood pressure measurements were performed in conscious rats.

**Results:**

Labda-15-oic acid (0.1-300 *µ*mol/l) and forskolin (0.1 nmol/l - 1 *µ*mol/l) relaxed endothelium-intact and endothelium-denuded aortas from both 2K-1C and 2K rats. Labda-15-oic acid was more effective at inducing relaxation in endothelium-intact aortas from 2K pre-contracted with phenylephrine when compared to the endothelium-denuded ones. Forskolin was more potent than labda-15-oic acid at inducing vascular relaxation in arteries from both 2K and 2K-1C rats. Labda-15-oic acid-induced increase in NOx levels was lower in arteries from 2K-1C rats when compared to 2K rats. Intravenous administration of labda-15-oic acid (0.3-3 mg/kg) or forskolin (0.1-1 mg/kg) induced hypotension in conscious 2K-1C and 2K rats.

**Conclusion:**

The present findings show that labda-15-oic acid induces vascular relaxation and hypotension in hypertensive rats.

## Introduction

The treatment of arterial hypertension with plant-derived products is well described in the literature.^1-4^ A great number of medicinal plants with antihypertensive activity have been chemically investigated and diterpenoids are pointed out as their major constituents. For this reason, many studies have focused on the cardiovascular properties of these compounds. For example, the labdane-type diterpene forskolin (7 beta-acetoxy-8, 13-epoxy-1 alpha,6 beta,9 alpha-trihydroxy-labd-14-ene-11-one) lowers blood pressure by a mechanism that involves relaxation of vascular smooth muscle.^5-8^ In the vasculature, forskolin activates the enzyme adenylyl cyclase, which in turn increases the production of cAMP and cAMP-dependent protein kinase (PKA) activation.^9^ Calcium extrusion across the plasma membrane and vascular smooth muscle hyperpolarization are mechanisms also related to the vascular actions of forskolin^10^. In humans, intravenous administration of forskolin decreased vascular resistance and reduced diastolic blood pressure (DBP).^7,8^

Other labdane-type diterpenes, such as labdane 8(17), 12E, 14-labdatrien-18-oic acid and labd-8 (17)-en-15-oic acid were also described to induce vascular relaxation and hypotension in normotensive rats.^11,12^ We have recently described that the labdane ent-3-acetoxy-labda-8(17),13-dien-15-oic acid (labda-15-oic acid) induced vascular relaxation via blockade of Ca^2+^ influx, activation of the endothelial nitric oxide (NO)-cGMP pathway and the opening of K^+^ channels.^13^ Intravenous injection of labda-15-oic acid induced a decrease in blood pressure in normotensive rats and this response was partially attenuated by L-NAME, suggesting a role for NO in such response.^13^ It is important to note that lower doses of labda-15-oic acid (0.3 - 3 mg/kg) were needed to induce hypotension when compared to other labdanes previously tested, such as 8 (17), 12E, 14-labdatrien-18-oic acid (5-30 mg/kg)^11^ and labd-8 (17)-en-15-oic acid (1-10 mg/kg).^12^ On the basis of these initial results with labda-15-oic acid, we hypothesized that this compound would induce vascular relaxation and hypotension in hypertensive rats. In the present study we sought to evaluate the cardiovascular actions of labda-15-oic acid in hypertensive animals.

## Methods

### Isolation of labda-15-oic acid

The isolation of labda-15-oic acid was performed as previously described.^14^ One hundred grams of oleoresin was chromatographed over silica gel 60 H (Merck, art. 7736) using vacuum liquid chromatography (VLC) with increasing amounts of ethyl acetate (EtOAc) in n-hexane as eluent. This procedure furnished six fractions (2000 ml each) that were named F1 (34.7 g; n-hexane), F2 (13.5 g; 20% EtOAc), F3 (11.4 g; 40% EtOAc), F4 (9.7 g; 60% EtOAc), F5 (7.6 g; 80% EtOAc), and F6 (17.8 g; EtOAc) after solvent evaporation. Fraction F4 was initially chromatographed by VLC over silica gel 60 H (Merck, art. 7736) as described above, to give additional fractions (F4.1 to F4.5). Labda-15-oic acid (1132.0 mg) was obtained from F4.3 through medium pressure chromatography (flash chromatography) using silica gel 60 (Merck, art. 9385), isocratic n-hexane: EtOAc:CHCl_3_ (5:2:3) as mobile phase, and a flow rate of 5 ml/min.^15^ The purity of (-)-acetoxycopalic acid (98%) was estimated by HPLC, mass spectrometric analysis and ^1^H and ^13^C NMR spectral data.

### Renovascular hypertension

Renovascular hypertension was induced in rats as previously described. Briefly, male Wistar rats weighting between 180 and 200 g (35 days old) were anaesthetised with tribromoethanol (250 mg/kg, i.p.) and after a midline laparotomy, a silver clip with an internal diameter of 0.2 mm was placed around the left renal artery. Normotensive two kidney (2K) rats were submitted to laparotomy only. Systolic blood pressure (SBP) was measured before and after 6 weeks of midline laparotomy in non anaesthetized animals by pletysmography (tail-cuff) and rats were considered to be hypertensive when SBP was higher than 160 mmHg. At 6 weeks after surgery, rats were killed and the thoracic aortas were isolated.^16^ A total of 26 2K rats and 28 2K-1C rats were used in the present study. All protocols were approved by the Ethical Animal Committee of the Campus of Ribeirão Preto - University of São Paulo (#09.1.1007.53.0).

### Vessel ring preparation

The thoracic aorta was quickly removed, cleaned of adherent connective tissues and cut into rings (5-6 mm in length). Two stainless-steel stirrups were passed through the lumen of each ring. One stirrup was connected to an isometric force transducer (TRI201; Panlab, Spain) to measure tension in the vessels. The rings were placed in a 5 ml organ chamber that contained Krebs solution, gassed with 95% O_2_ / 5% CO_2_ maintained at 37°C. The composition of Krebs solution was as follows (mmol/l): NaCl, 118.0; KCl, 4.7; KH_2_PO_4_, 1.2; MgSO_4_, 1.2; NaHCO_3_, 15.0; Glucose, 5.5; CaCl_2_, 2.5. The rings were stretched until they reached a basal tension of 1.5 g, which was determined by length-tension relationship experiments and were then allowed to equilibrate for 60 min; during this time, the bath fluid was changed every 15-20 min. For some rings, the endothelium was removed mechanically by gently rolling the lumen vessel on a thin wire. Endothelial integrity was assessed qualitatively by the degree of relaxation caused by acetylcholine (1 *µ*mol/l) in the presence of contractile tone induced by phenylephrine (0.1 *µ*mol/l). For studies of endothelium-intact vessels, a ring was discarded if relaxation with acetylcholine was not 50% or greater. For studies of endothelium-denuded vessels, a ring was discarded if there was any degree of relaxation. Agonist concentration-response curves were fitted using a nonlinear interactive fitting program (Graph Pad Prism 3.0; GraphPad Software Inc., San Diego, CA, USA). Agonist potencies and maximal responses were expressed as pD_2_ (-logEC_50_) and Emax (maximum effect elicited by the agonist), respectively.

### Effect of labda-15-oic acid on aortic rings contracted with phenylephrine or KCl

Steady tension was evoked by phenylephrine (concentrations of 0.1 *µ*mol/l for endothelium-intact rings and 0.03 *µ*mol/l for endothelium-denuded rings were used to induce contractions of similar magnitude), and labda-15-oic acid was then added in a stepwise fashion (0.1-300 *µ*mol/l). The effect of labda-15-oic acid on KCl-induced sustained contraction (30 mmol/l) in intact or denuded rings was also examined. For comparison, the effect of forskolin (0.1 nmol/l - 1 *µ*mol/l) on the contractions induced by phenylephrine and KCl in endothelium-intact and endothelium-denuded rings was evaluated.

### Blood pressure experiments

Blood pressure experiments were performed as previously described.^17^ One day before the experiments, the rats were anesthetised with tribromoethanol (250 mg/kg, i.p.), and a catheter (a 4 cm segment of PE-10 heat-bound to a 13 cm segment of PE-50 (Clay Adams, Parsippany, NJ, USA) was inserted into the abdominal aorta through the femoral artery for blood pressure and heart rate recording. A second catheter was implanted into the jugular vein for intravenous administration of drugs. Both catheters were implanted under the skin and exited at the animal's back. During the experiment, freely moving rats were kept in individual cages, and mean arterial pressure (MAP) was recorded using an HP-7754A amplifier (Hewlett Packard, USA) connected to a signal acquisition board (MP-100, BIOPAC, USA) and processed by a computer. Labda-15-oic acid (0.3 - 3 mg/kg) or forskolin (0.1 - 1 mg/kg) were administered by intravenous bolus injection. Both labda-15-oic acid (0.3-3 mg/kg) and forskolin (0.1 - 1 mg/kg) were administered in different animals. Blood pressure responses were calculated with base on the average mean blood pressure calculated at the response's plateau.

### Nitrate/Nitrite (NOx) measurements

NOx levels were measured in supernatants from endothelium-intact aorta homogenates from 2K-1C and 2K rats. The rings were pre-contracted with phenylephrine (0.1 *µ*mol/l) and then exposed to labda-15-oic acid (300 *µ*mol/l). Supernatants were centrifuged using ultra centrifugal filters (#UFC5010BK Amicon Ultra-0.5 mL 10 kDa, Millipore, Billerica, MA, USA). Nitrate was measure colorimetrically following the instructions of a commercially available kit (#780,001, Cayman Chemical, Ann Arbor, MI, USA). Results were normalized for protein concentration and are expressed as nmol/mg protein. Protein concentrations in all experiments were determined (with protein assay reagent (Bio-Rad Laboratories, Hercules, CA, USA).

### Drugs

Labda-15-oic acid was prepared as stock solutions in dimethyl sulfoxide (DMSO). The other drugs were dissolved in distilled water. The bath concentration of DMSO did not exceed 0.5%, which was shown to have no effect per se on the basal tonus of the preparations or on the agonist-mediated contraction or relaxation. For the in vivo experiments, labda-15-oic acid was diluted in 10% DMSO and then in saline. The concentration of DMSO in the final solution had no effects per se on basal cardiovascular parameters, as previously observed.^18^

### Statistical analysis

Results were expressed as means standard error of the mean (S.E.M.). Data followed a normal distribution. Statistical analysis was performed using one-way analysis of variance (ANOVA) or paired Student's t test. Post-hoc comparisons were performed after ANOVA analysis using Newman-Keuls multiple comparison test as indicated in the text and tables. For all analyses, p values of less than 0.05 were considered significant. Statistical analysis was carried out using the program Graph Pad Prism 3.0 (GraphPad Software Inc., San Diego, CA, USA).

## Results

### Blood pressure values in 2K-1C and 2K rats

MAP, DBP and SBP were significantly increased in 2K-1C when compared to 2K rats (Table 1).

**Table 1:** Blood pressure values (mmHg) in 2K and 2K-1C rats

	2K		2K-1C	
	Basal	After 6 weeks	Basal	After 6 weeks
MAP	104.3 ± 2.0	100.9 ± 1.6	105.7 ± 1.1	161.3 ± 10.4^a^
DBP	92.5 ± 1.8	89.8 ± 1.3	96.3 ± 1.1	138.4 ± 11.6^a^
SBP	127.9 ± 2.8	123.2 ± 2.9	124.6 ± 1.9	207.0 ± 9.2^a^

Values are means ± S.E.M of n = 12 animals for each group.

a Compared to respective basal values (p < 0.05, paired Student's t test). MAP: mean arterial pressure; DBP: diastolic blood pressure; SBP: systolic blood pressure.

### Vasorelaxant action of labda-15-oic acid on aortic rings from 2K-1C and 2K rats

**Figure 1:**
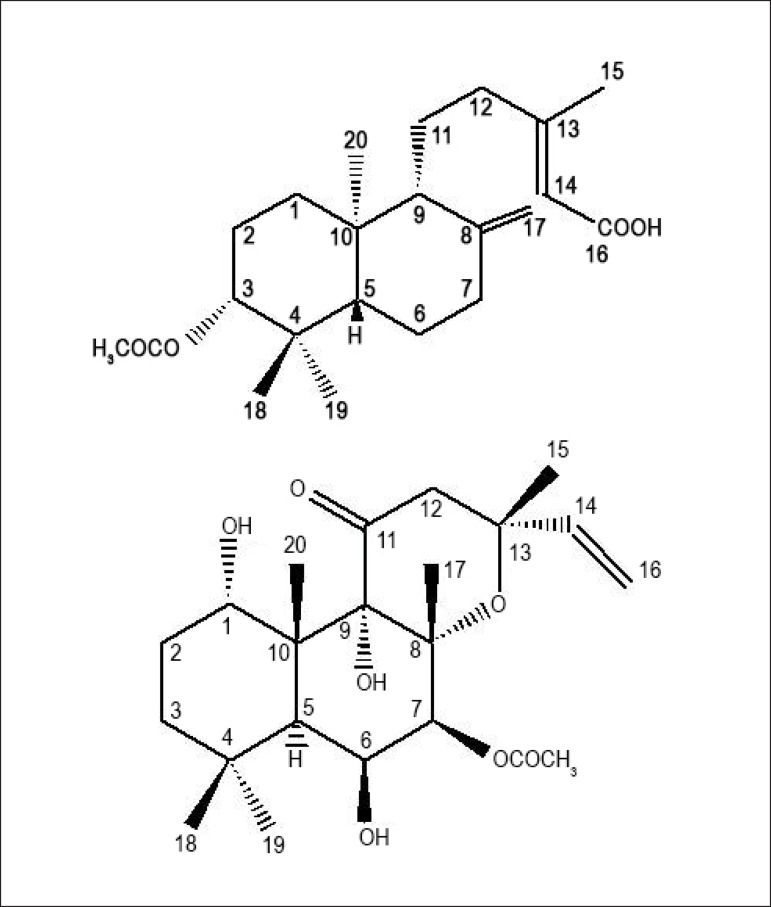
Chemical structure of ent-3-acetoxy-labda-8(17),13-dien- 15-oic acid (labda-15-oic acid; top) and 7 beta-acetoxy-8, 13-epoxy-1 alpha,6 beta,9 alpha-trihydroxy-labd-14-ene-11-one (forskolin, bottom).

**Figure 2:**
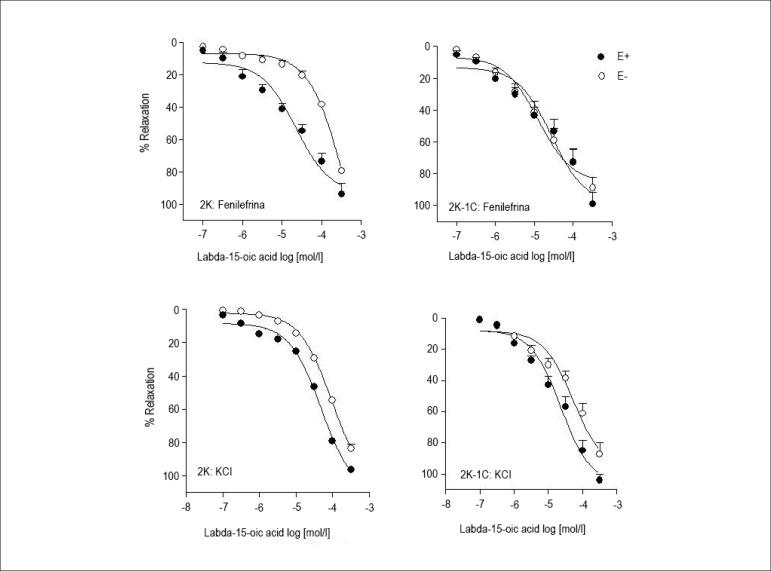
Relaxation responses induced by labda-15-oic acid on rat aortic rings. The relaxation induced by the labdane was studied on endothelium-intact (E+) and endothelium-denuded (E-) rat aortic rings contracted with either phenylephrine (0.1 µmol/l) or KCl (30 mmol/l). Steady tension was evoked by phenylephrine or KCl and then labda-15-oic acid (0.1 - 300 µmol/l) was added cumulatively.

Labda-15-oic acid (Figure 1) reduced the sustained contractions induced by phenylephrine and KCl in endothelium-intact and endothelium-denuded aortas from both 2K-1C and 2K rats (Figure 2). The E_max_ values (percentage of relaxation) for the relaxant effect of labda-15-oic acid in endothelium-intact and endothelium-denuded rings pre-contracted with phenylephrine were not significantly different in aortas from 2K-1C and 2K rats (Table 2). However, differences were found in the pD_2_ values for labda-15-oic acid in endothelium-intact and denuded rings pre-contracted with phenylephrine in aortas from 2K, but not 2K-1C rats. In the arteries pre-contracted with KCl, there was no difference between the E_max_ and pD_2_ values for labda-15-oic acid in endothelium-intact or denuded rings from both 2K-1C and 2K rats (Table 2). The E_max_ and pD_2_ values for labda-15-oic acid in the rings pre-contracted with KCl were not different from those found in phenylephrine-pre-contracted rings from both 2K-1C and 2K rats.

**Table 2:** E_max_ (% relaxation) and pD_2_ values for labda-15-oic acid and forskolin in endothelium-intact (E+) and endothelium-denuded (E-) aortas from 2K and 2K-1C rats

	Pre-contractile agent	2K		2K-1C	
		E+ (E_max_)	E- (E_max_)	E+ (E_max_)	E- (E_max_)
Labda-15-oic acid	Phenylephrine	93.7 ± 6.8 (7)	79.2 ± 1.8 (6)	99.0 ± 7.4 (7)	88.8 ± 6.6 (6)
	KCl	96.4 ± 4.4 (7)	83.6 ± 6.6 (6)	103.9 ± 3.8 (7)	87.3 ± 7.4 (8)
Forskolin	Phenylephrine	110.7 ± 5.3 (7) ^a^	104.0 ± 5.62a (6)	118.8 ± 5.2 (6) ^a^	107.7 ± 8.0 (6) ^a^
	KCl	92.6 ± 3.9 (6)	87.8 ± 3.9 (5)	105.9 ± 3.3 (6)	93.2 ± 7.1 (6)
		**E+ (pD_2_)**	**E- (pD_2_)**	**E+ (pD_2_)**	**E- (pD_2_)**
Labda-15-oic acid	Phenylephrine	4.8 ± 0.06 (7)	4.1 ± 0.04 (6)^b^	4.8 ± 0.11 (7)	4.9 ± 0.08(6)
	KCl	4.6 ± 0.08 (7)	4.3 ± 0.06 (6)	4.8 ± 0.10 (7)	4.5 ± 0.08 (8)
Forskolin	Phenylephrine	7.5 ± 0.21 (7) ^c^	6.9 ± 0.17(6) ^b,c^	8.0 ± 0.10 (6) ^c^	7.3 ± 0.14(6) ^b,c^
	KCl	7.0 ± 0.16 (6) ^c^	7.0 ± 0.15(5) ^c^	7.3 ± 0.20 (6) ^c^	7.0 ± 0.12 (6) ^c^

Numbers within parentheses indicate the number of isolated preparations. Values are means ± S.E.M.

a Compared to labda-15-oic acid in aortas pre-contracted with phenylephrine from 2K and 2K-1C rats;

b Compared to respective group in E+ aortas from 2K and 2K-1C rats;

c Compared to labda-15-oic acid in aortas pre-contracted with phenylephrine or KCl from 2K and 2K-1C rats (p < 0.05, ANOVA followed by Newman-Keuls multiple comparison test).

**Figure 3:**
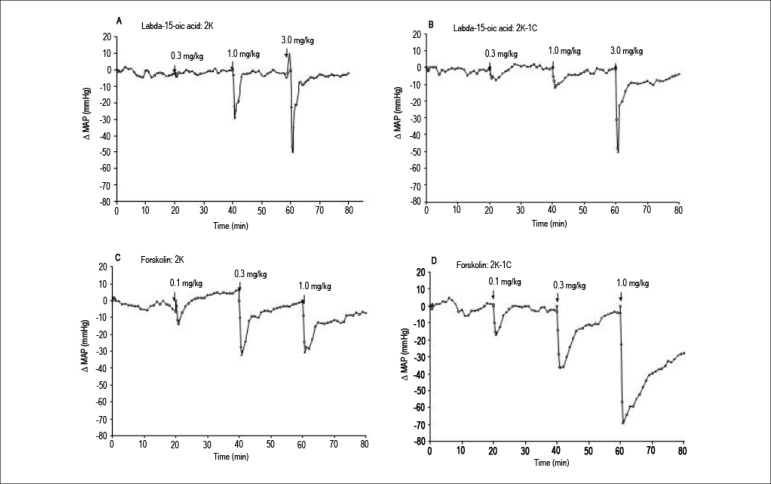
Relaxation responses induced by forskolin on rat aortic rings. The relaxation induced by the labdane was studied on endothelium-intact (E+) and endothelium‑denuded (E-) rat aortic rings contracted with either phenylephrine (0.1 µmol/l) or KCl (30 mmol/l). Steady tension was evoked by phenylephrine or KCl and then forskolin (0.1 nmol/l - 1 µmol/l) was added cumulatively.

Forskolin reduced the sustained contractions induced by phenylephrine and KCl in endothelium-intact and endothelium-denuded aortas from both 2K-1C and 2K rats (Figure 3). The E_max_ values for the relaxant effect of forskolin in endothelium-intact and endothelium-denuded rings pre-contracted with phenylephrine were not significantly different in aortas from 2K-1C and 2K rats (Table 2). However, differences were found in the pD_2_ values for forskolin in endothelium-intact and denuded rings pre-contracted with phenylephrine in aortas from both 2K-1C and 2K rats. In the arteries pre-contracted with KCl, there was no difference between the E_max_ or pD_2_ values for forskolin in endothelium-intact or denuded rings from both 2K-1C and 2K rats (Table 2).

The E_max_ values for forskolin in endothelium-intact and endothelium-denuded rings pre-contracted with phenylephrine, but not KCl, were significantly different from those found for labda-15-oic acid in both 2K-1C and 2K rats. The pD_2_ values for forskolin in endothelium-intact and denuded rings pre-contracted with either phenylephrine or KCl were significantly different from those found for labda-15-oic acid in both 2K-1C and 2K rats (Table 2).

### Blood pressure experiments

**Figure 4:**
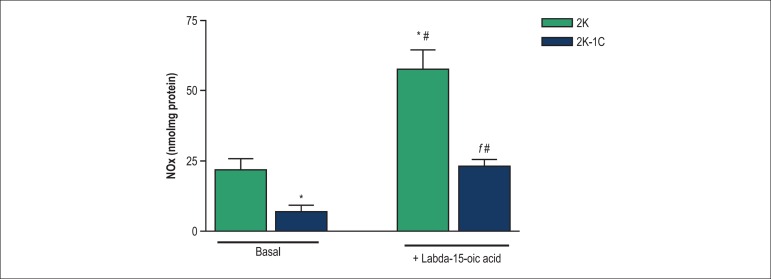
Representative traces of the hypotensive action displayed by labda-15-oic acid (0.3 – 3 mg/kg) and forskolin (0.1 – 1 mg/kg) on conscious 2K and 2K-1C rats. Traces represent the mean values of the maximal decrease in mean arterial pressure of 5 to 6 animals.

**Figure 5:**
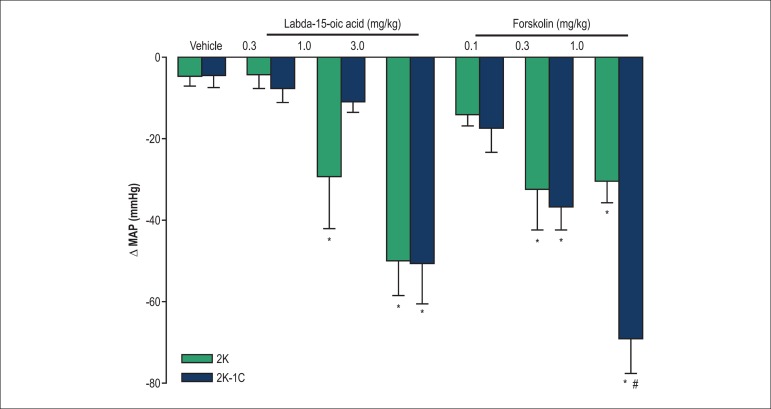
Effect of labda-15-oic acid (0.3 – 3 mg/kg) and forskolin (0.1 – 1 mg/kg) on mean arterial pressure (MAP). Maximal variation in MAP (mmHg) induced by intravenous injection of the labdanes was evaluated in conscious 2K and 2K-1C rats. Each bar represents the mean ± S.E.M. of 5 to 6 experiments. *Compared with vehicle; #Compared with 2K rats (p < 0.05, ANOVA followed by Newman-Keuls multiple comparison test).

Figure 4 shows representative tracings for the effect of labda-15-oic acid and forskolin on blood pressure of 2K and 2K-1C rats. The maximal variation in MAP induced by labda-15-oic acid and forskolin in conscious 2K-1C and 2K rats is presented in Figure 5. A bolus injection of labda-15-oic acid or forskolin produced a decrease in MAP in conscious 2K-1C and 2K rats. The MAP values returned to basal levels after injection of labda-15-oic acid. On the other hand, MAP values did not return to basal levels after administration of forskolin at 1 mg/kg (Figure 5). Labda-15-oic acid induced a more pronounced fall in blood pressure in 2K when compared to 2K-1C rats. On the other hand, forskolin was found to be more effective at inducing decrease in MAP in 2K-1C when compared to 2K rats (Figure 5). Values of blood pressure before and after drug administration are described in Table 3.

**Table 3:** Blood pressure values (mmHg) in 2K and 2K-1C rats before and after drug administration (labda-15-oic acid or forskolin) and its respective values ΔMAP and %ΔMAP

	MAP (mmHg)			
	Before	After	ΔMAP	%ΔMAP
**Labda-15-oic acid 2K**				
Vehicle	103.5 ± 6.7 (5)	98.9 ± 7.0	4.6 ± 2.4	4.4 ± 2.3
Labda-15-oic acid (0.3 mg/kg)	100.5 ± 5.5 (5)	96.3 ± 8.4	4.2 ± 3.3	4.8 ± 3.8
Labda-15-oic acid (1 mg/kg)	99.8 ± 6.2 (5)	70.6 ± 15.6	29.2 ± 12.7	30.3 ± 13.6
Labda-15-oic acid (3 mg/kg)	98.5 ± 6.4 (5)	48.6 ± 12.4^a^	49.9 ± 8.5^b^	53.6 ± 10.9 ^b^
**Labda-15-oic acid 2K-1C**				
Vehicle	163.7 ± 15.2 (6)	159.3 ± 16.2	4.4 ± 3.0	3.0 ± 2.3
Labda-15-oic acid (0.3 mg/kg)	161.6 ± 15.8 (6)	154.0 ± 16.5	7.6 ± 3.3	5.2 ± 2.5
Labda-15-oic acid (1 mg/kg)	160.0 ± 15.6 (6)	148.0 ± 15.7	12.0 ± 3.6	7.9 ± 2.7
Labda-15-oic acid (3 mg/kg)	160.2 ± 15.8 (6)	109.7 ±19.2^a^	50.5 ± 9.9^b^	33.8 ± 8. 4^b^
**Forskolin 2K**				
Vehicle	113.9 ± 3.0 (5)	107.5 ± 4.3	6.4 ± 1.7	5.7 ± 1.5
Forskolin (0.1 mg/kg)	104.9 ± 4.5 (5)	90.9 ± 5.2^a^	14.0 ± 2.7	13.4 ± 2.8
Forskolin (0.3 mg/kg)	108.1 ± 5.0 (5)	75.8 ± 10.8^a^	32.3 ± 10.0^b^	29.9 ± 9.6^b^
Forskolin (1 mg/kg)	107.4 ± 4.0 (5)	77.0 ± 3.1^a^	30.4 ± 5.2^b^	27.9 ± 3.8^b^
**Forskolin 2k-1C**				
Vehicle	169.1 ± 12.8 (5)	163.3 ± 15.1	5.8 ± 4.6	3.7 ± 2.5
Forskolin (0.1 mg/kg)	170.4 ± 16.6 (5)	153.2 ± 12.9^a^	17.2 ± 6.1	9.4 ± 3.1
Forskolin (0.3 mg/kg)	167.6 ± 16.3 (5)	130.9 ± 12.4^a^	36.7 ± 5.7^b^	21.7 ± 2.5^b^
Forskolin (1 mg/kg)	166.0 ± 16.9 (5)	97.1 ± 16.0^a^	68.9 ± 8.5^b^	42.4 ± 6.2^b^

Numbers within parentheses indicate the number of animals. Values are means ± S.E.M.

a Significant difference compared to baseline, before drug infusion (p < 0.05, paired Student’s t test).

b Compared with vehicle (p < 0.05, ANOVA followed by Newman-Keuls multiple comparison test). MAP: mean arterial pressure.

### NOx measurements

**Figure 6:**
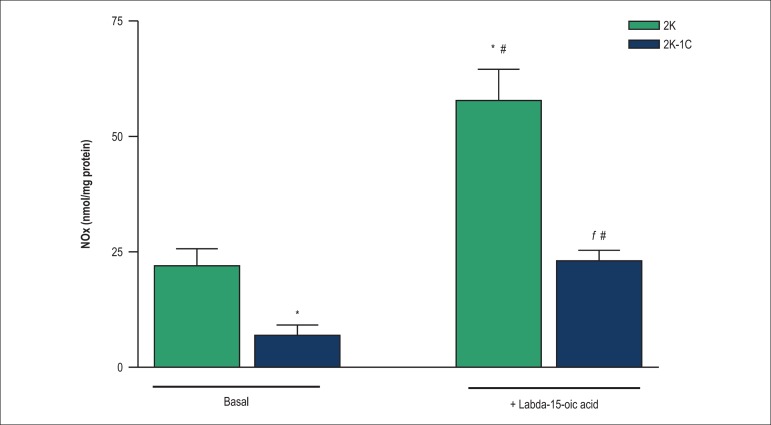
Effect of labda-15-oic acid on nitrate levels in endothelium-intact aortic rings from 2K and 2K-1C rats. Each bar represents the mean ± S.E.M. of 6 to 8 independent preparations. *Compared with basal values for 2K rats; #Compared with basal values for 2K-1C rats; ^ƒ^Compared with stimulation with labda-15-oic acid in 2K-1C rats (p < 0.05, ANOVA followed by Newman-Keuls multiple comparison test).

Figure 6 show that NOx basal levels in aortas from 2K-1C rats are lower than those found in aortas from 2K rats. Labda-15-oic acid induced nitrate generation in endothelium-intact aortas from both 2K-1C and 2K rats. Labda-15-oic acid-induced nitrate generation was lower in arteries from 2K-1C rats when compared to 2K rats (Figure 6).

## Discussion

The present findings show that labda-15-oic acid was more effective at inducing vascular relaxation in endothelium-intact aortas from 2K rats pre-contracted with phenylephrine when compared to the endothelium-denuded ones. This result is in accordance with previous finding from our laboratory showing that the relaxation induced by labda-15-oic acid is partially dependent on the endothelial cGMP-NO pathway.^13^ On the other hand, in aortas from 2K-1C rats, no difference on labda-15-oic acid-induced relaxation was observed between endothelium-intact and denuded rings. Altered vascular tone is a characteristic feature of most forms of experimental and human hypertension and has been associated with endothelial dysfunction with consequent impairment of endothelium-dependent vasodilatation and reduced NO signalling.^19-21^ Since endothelial-derived NO partially mediates the vasorelaxant effect of labda-15-oic acid, the decrease in potency for the relaxant action of the labdane in aortas from 2K-1C rats might be due to the decreased NO bioavailability described in hypertensive states. In fact, this hypothesis is strengthened by the fact that labda-15-oic acid-induced nitrate generation in arteries from 2K-1C was lower than that found in arteries from 2K rats. It is also important to note that we found lower basal NOx content in arteries from 2K-1C when compared to aortas from 2K rats, further corroborating previous observations showing decreased availability of basal NO in renovascular hypertension.^22-24^

The activation of K^+^ channels leads to hyperpolarization of vascular smooth muscle cells, decrease in voltage-dependent Ca^2+^ channel activity, and vasodilatation.^25^ The activation of voltage-dependent and ATP-sensitive K^+^ channels, as well as large-conductance and low-conductance Ca^2+^-activated K^+^ channels was described to play a role in the vasorelaxant response induced by labda-15-oic acid^13^. It is well established that endothelium-dependent vasodilatation and smooth muscle cell hyperpolarization are impaired in aortic segments from 2K-1C hypertensive rats.^26^ Abnormal function of vascular smooth muscle large-conductance Ca^2+^-activated K^+^ channels and ATP-sensitive K^+^ channels play a key role in the impaired relaxation of aortas from 2K-1C rats,^27,28^ and may also contribute to the decreased endothelium-dependent vasodilatation induced by labda-15-oic acid in aortas from 2K-1C rats.

In the present study, no differences were found in the inhibitory action displayed by labda-15-oic acid in arteries pre-contracted with KCl in both 2K and 2K-1C rats. The contraction induced by KCl on smooth muscle is mediated by cell membrane depolarisation and an increase in Ca^2+^ influx through voltage-operated Ca^2+^ channels.^29,30^ Thus, we can suggest that labda-15-oic acid blocks extracellular Ca^2+^ influx through interference with voltage-operated channels in 2K and 2K-1C rats.

Forskolin relaxed endothelium-intact and endothelium-denuded aortas pre-contracted with phenylephrine, but not KCl, to a greater extent than labda-15-oic acid in both 2K and 2K-1C rats. Moreover, forskolin was more potent than labda-15-oic acid at inducing vascular relaxation in arteries pre-contracted with phenylephrine or KCl in both 2K and 2K-1C rats. Possible explanations for these effects are related to the chemical structure of the labdanes and/or their mechanisms of action. Analyzing the chemical structure of labda-15-oic acid and forskolin (Figure 1) we observe that, despite the fact that these two compounds are classified as labdane type-diterpenes, it is noteworthy the presence of great number of hydrogen-bond-donor groups (HBD; hydrophilic group), highlighting the hydroxyl moieties at C-1, C-6 and C-9, in the forskolin skeleton in comparison with the chemical structure of labda-15-oic acid, which contains only two hydrophilic groups at C-3 and C-16. Moreover, it is also possible to observe that these natural compounds differ from each other in their inverted configurations of the carbons C-5, C-9 and C-10. Previous studies have shown that chemical differences on diterpenes alter their cardiovascular properties,^17,31^ and might be the source of discrepancy between the effects of labda-15-oic acid and forskolin here described.

Labdanes exert their cardiovascular effects by acting at multiple sites,^11,12,32^ and for this reason, several intracellular pathways were described to mediate the vascular relaxation induced by these compounds.^33^ The increase in cAMP levels, due to activation of adenylyl cyclase and the subsequent activation of PKA is the main mechanism underlying the vascular relaxation induced by the labdane forskolin.^9^ However, forskolin also increases endothelial production of NO via activation of eNOS.^34^ On the other hand, the mechanisms underlying the vasorelaxant action of labda-15-oic acid are not related to adenylyl cyclase activation and involve blockage of extracellular Ca^2+^ influx, increased endothelial NO production and the opening of K^+^ channels.^13^ The differences in the mechanisms underlying the vascular responses of these two labdanes could also be responsible for the different cardiovascular responses displayed by labda-15-oic acid and forskolin.

Improvements in the pharmacological treatment of hypertension contribute to a reduction in the incidence of cardiovascular diseases.^35^ Labdane-type diterpenes could be considered a promising source of new prototypes for the discovery and development of novel cardiovascular therapeutic agents. The hypotensive action of labdane-type diterpenes is related to their myorelaxant action.^5,6,11,12^ Recently, we described that labda-15-oic acid induces vascular relaxation and hypotension in normotensive rats.^13^ Since labda-15-oic acid relaxed aortas from 2K-1C rats, we hypothesized that the labdane could exert antihypertensive action in vivo. In the present study, intravenous administration of labda-15-oic acid induced a short-lasting hypotension in 2K and 2K-1C rats, further showing that labda-15-oic acid exert antihypertensive effect in vivo. Labda-15-oic acid induced a less pronounced decrease in blood pressure compared to forskolin, further strengthening the idea that chemical differences alters the hypotensive action displayed by labdane-type diterpenes. It is also important to note that labda-15-oic acid causes hypotension through peripheral vasodilatation, mediated in part by NO,^13^ while forskolin effects are mainly mediated by activation of adenylate cyclase and the increase in cAMP levels.^5-9^ This observation is relevant since, as mentioned before, endothelial dysfunction with consequent impairment of endothelium-dependent vasodilatation and reduced NO signalling is a characteristic feature of hypertension.^19-21^ This characteristic of the hypertensive state could explain, at least in part, the reduced effect of labda-15-oic acid in comparison to forskolin.

Some limitations for the present study should be considered. Despite the fact that labda-15-oic acid decreased blood pressure in an animal model of renovascular hypertension, it is not possible to guarantee that this labdane will be also effective on other animal models of hypertension or human hypertension. Another point that should be considered is that the vasorelaxant effect of the labdane should also be tested in resistance vessels since those are more important in the regulation of blood pressure. Finally, our findings show the effects of labda-15-oic acid after intravenous injection of the compound but we do not have information on the bioavailability and cardiovascular effects of this compound after oral administration.

## Conclusions

Diterpenes likely fulfill the definition of a pharmacological preconditioning class of compounds and may have therapeutic use in cardiovascular diseases. Using a combined in vivo and in vitro approach, the present investigation shows for the first time that labda-15-oic acid induces vascular relaxation in arteries from 2K-1C hypertensive rats. Administration of the labdane in vivo induced a fall in blood pressure in hypertensive rats. The initial experimental studies on the cardiovascular effects of labdanes are important and needed, since such information is a prerequisite to any rational and safety use of these compounds in the treatment of hypertension.

## References

[1] Alonso A, Martínez-González MA (2004). Olive oil consumption and reduced incidence of hypertension: the
SUN study. Lipids.

[2] Herrera-Arellano A, Flores-Romero S, Chávez-Soto MA, Tortoriello J (2004). Effectiveness and tolerability of a standardized extract from
Hibiscus sabdariffa in patients with mild to moderate hypertension: a
controlled and randomized clinical trial. Phytomedicine.

[3] Herrera-Arellano A, Miranda-Sánchez J, Avila-Castro P, Herrera-Alvarez S, Jiménez-Ferrer JE, Zamilpa A (2007). Clinical effects produced by a standardized herbal medicinal
product of Hibiscus sabdariffa on patients with hypertension.A randomized,
double-blind, lisinopril-controlled clinical trial. Planta Med.

[4] McKay DL, Diane L, Oliver Chen CY, Saltzman E, Blumberg JB (2010). Hibiscus sabdariffa L. tea (tisane) lowers blood pressure in
prehypertensive and mildly hypertensive adults. J Nutr.

[5] Lindner E, Dohadwalla AN, Bhattacharya BK (1978). Positive inotropic and blood pressure lowering activity of a
diterpene derivative isolated from Coleus forskoli:
Forskolin. Arzneimittelforschung.

[6] Dubey MP, Srimal RC, Nityanand S, Dhawan BN (1981). Pharmacological studies on coleonol, a hypotensive diterpene from
Coleus forskohlii. J. Ethnopharmacol.

[7] Kramer W, Thormann J, Kindler M, Schlepper M (1987). Effects of forskolin on left ventricular function in dilated
cardiomyopathy. Arzneimittelforschung.

[8] Schlepper M, Thormann J, Mitrovic V (1989). Cardiovascular effects of forskolin and phosphodiesterase-III
inhibitors. Basic Res Cardiol.

[9] Lincoln TM, Fisher-Simpson V (1984). A comparison of the effects of forskolin and nitroprusside on
cyclic nucleotides and relaxation in the rat aorta. Eur J Pharmacol.

[10] Den Hertog A, Pielkenrood J, Van den Akker JT (1984). The effect of forskolin on smooth muscle cells of guinea-pig
taenia caeci. Eur J Pharmacol.

[11] de Oliveira AP, Furtado FF, da Silva MS, Tavares JF, Mafra RA, Araújo DA (2006). Calcium channel blockade as a target for the cardiovascular
effects induced by the 8 (17), 12E, 14-labdatrien-18-oic acid
(labdane-302). Vascul Pharmacol.

[12] Lahlou S, de Barros Correia CA, Vasconcelos dos Santos M, David JM, David JP, Duarte GP (2007). Mechanisms underlying the cardiovascular effects of a labdenic
diterpene isolated from Moldenhawera nutans in normotensive
rats. Vascul Pharmacol.

[13] Simplicio JA, Pernomian L, Simão MR, Carnio EC, Batalhão ME, Ambrosio SR (2014). Mechanisms underlying the vascular and hypotensive actions of the
labdane ent-3-acetoxy-labda-8(17),13-dien-15-oic acid. Eur. J. Pharmacol.

[14] Souza AB, de Souza MG, Moreira MA, Moreira MR, Furtado NA, Martins CH (2011). Antimicrobial evaluation of diterpenes from Copaifera
langsdorffii oleoresin against periodontal anaerobic
bacteria. Molecules.

[15] Still WC, Kahn M, Mitra A (1978). Rapid chromatographic technique for preparative separations with
moderate resolution. J Org Chem.

[16] Callera GE, Varanda WA, Bendhack LM (2001). Ca(2+) influx is increased in 2-kidney, 1-clip hypertensive rat
aorta. Hypertension.

[17] Hipólito UV, Rocha JT, Palazzin NB, Rodrigues GJ, Crestani CC, Corrêa FM (2011). The semi-synthetic kaurane ent-16a-methoxykauran-19-oic acid
induces vascular relaxation and hypotension in rats. Eur J Pharmacol.

[18] Tirapelli CR, Legros E, Brochu I, Honoré JC, Lanchote VL, Uyemura SA (2008). Chronic ethanol intake modulates vascular levels of endothelin-1
receptor and enhances the pressor response to endothelin-1 in anaesthetized
rats. Br J Pharmacol.

[19] Puddu P, Puddu GM, Zaca F, Muscari A (2000). Endothelial dysfunction in hypertension. Acta Cardiol.

[20] Schiffrin EL (2001). A critical review of the role of endothelial factors in the
pathogenesis of hypertension. J Cardiovasc Pharmacol.

[21] Taddei S, Salvetti A (2002). Endothelial dysfunction in essential hypertension: clinical
implications. J Hypertens.

[22] Heitzer T, Wenzel U, Hink U, Krollner D, Skatchkov M, Stahl RA (1999). Increased NAD(P)H oxidase-mediated superoxide production in
renovascular hypertension: evidence for an involvement of protein kinase
C. Kidney Int.

[23] Higashi Y, Sasaki S, Nakagawa K, Matsuura H, Oshima T, Chayama K (2002). Endothelial function and oxidative stress in renovascular
hypertension. N Engl J Med.

[24] Jung O, Schreiber JG, Geiger H, Pedrazzini T, Busse R, Brandes RP (2004). gp91phox-containing NADPH oxidase mediates endothelial
dysfunction in renovascular hypertension. Circulation.

[25] Nelson MT, Quayle JM (1995). Physiological roles and properties of K(+) channels in arterial
smooth muscle. Am J Physiol.

[26] Callera GE, Varanda WA, Bendhack LM (2000). Impaired relaxation to Ach in 2K-1C hypertensive rat aortas
involves changes in membrane hyperpolarization instead of an abnormal
contribution of endothelial factors. Gen Pharmacol.

[27] Callera GE, Yogi A, Tostes RC, Rossoni LV, Bendhack LM (2004). Ca(2+-activated K(+) channels underlying the impaired
acetylcholine-induced vasodilation in 2K-1C hypertensive
rats. J Pharmacol Exp Ther.

[28] Callera GE, Yeh E, Tostes RC, Caperuto LC, Carvalho CR, Bendhack LM (2004). Changes in the vascular beta-adrenoceptor-activated signalling
pathway in 2Kidney-1Clip hypertensive rats. Br J Pharmacol.

[29] Hudgins PM, Weiss GB (1968). Differential effects of calcium removal upon vascular smooth
muscle contraction induced by norepinephrine, histamine and
potassium. J Pharm Exp Ther.

[30] Somlyo AP, Somlyo AV (1994). Signal transduction and regulation in smooth
muscle. Nature.

[31] Tirapelli CR, Ambrosio SR, Coutinho ST, De Oliveira DC, Da Costa FB, De Oliveira AM (2005). Pharmacological comparison of the vasorelaxant action displayed
by kaurenoic acid and pimaradienoic acid. J Pharm Pharmacol.

[32] El Bardai S, Wibo M, Hamaide M.C, Lyoussi B, Quetin-Leclercq J, Morel N (2003). Characterisation of marrubenol, a diterpene extracted from
Marrubium vulgare, as an L-type calcium channel blocker. Br J Pharmacol.

[33] Tirapelli CR, Ambrosio SR, de Oliveira AM, Tostes RC (2010). Hypotensive action of naturally occurring diterpenes: a
therapeutic promise for the treatment of hypertension. Fitoterapia.

[34] Anjos M, Lunardi CN, Rodrigues GJ, Bendhack LM (2011). Vasodilatation induced by forskolin involves cyclic GMP
production. J Biophysical Chem.

[35] Campbell NR, Brant R, Johansen H, Walker RL, Wielgosz A, Onysko J, Canadian Hypertension Education Program Outcomes Research Task
Force (2009). Increases in antihypertensive prescriptions and reductions in
cardiovascular events in Canada. Hypertension.

